# University students with attention deficit hyperactivity disorder (ADHD): a consensus statement from the UK Adult ADHD Network (UKAAN)

**DOI:** 10.1186/s12888-022-03898-z

**Published:** 2022-04-22

**Authors:** Jane A. Sedgwick-Müller, Ulrich Müller-Sedgwick, Marios Adamou, Marco Catani, Rebecca Champ, Gísli Gudjónsson, Dietmar Hank, Mark Pitts, Susan Young, Philip Asherson

**Affiliations:** 1 Health and Community Services, Government of Jersey, St Helier, Jersey. Social, Genetic & Developmental Psychiatry, Institute of Psychiatry, Psychology & Neuroscience (IoPPN) & Florence Nightingale Faculty of Nursing, Midwifery & Palliative Care (FNFNM), King’s College London, London, UK; 2 Adult Neurodevelopmental Service, Health and Community Services, Government of Jersey, St Helier, Jersey. Department of Psychiatry, University of Cambridge, Cambridge, UK; 3 School of Human and Health Sciences, University of Huddersfield, Huddersfield, UK; 4 Natbrainlab, Forensic and Neurodevelopmental Sciences, Institute of Psychiatry, Psychology & Neuroscience (IoPPN), King’s College London, London, UK; 5 Psychology Department, Institute of Psychiatry, Psychology & Neuroscience (IoPPN), King’s College London, London, UK; 6 Adult ADHD Service, Avon and Wiltshire Mental Health Partnership NHS Trust, Bristol, UK; 7 Adult ADHD and Autism Outpatient Service, South London & Maudsley NHS Foundation Trust, London, UK; 8 Psychology Services Limited, Department of Psychology, Reykjavik University, Reykjavik, Iceland; 9 Social, Genetic & Developmental Psychiatry, Institute of Psychiatry, Psychology & Neuroscience (IoPPN), King’s College London, London, UK

**Keywords:** Attention-deficit/hyperactivity disorder, ADHD, Academic performance, Academic achievement, University students, Educational outcomes, University, College, Higher education

## Abstract

**Background:**

Attention deficit hyperactivity disorder (ADHD) is associated with poor educational outcomes that can have long-term negative effects on the mental health, wellbeing, and socio-economic outcomes of university students. Mental health provision for university students with ADHD is often inadequate due to long waiting times for access to diagnosis and treatment in specialist National Health Service (NHS) clinics. ADHD is a hidden and marginalised disability, and within higher education in the UK, the categorisation of ADHD as a specific learning difference (or difficulty) may be contributing to this.

**Aims:**

This consensus aims to provide an informed understanding of the impact of ADHD on the educational (or academic) outcomes of university students and highlight an urgent need for timely access to treatment and management.

**Methods:**

The UK Adult ADHD Network (UKAAN) convened a meeting of practitioners and experts from England, Wales, and Scotland, to discuss issues that university students with ADHD can experience or present with during their programme of studies and how best to address them. A report on the collective analysis, evaluation, and opinions of the expert panel and published literature about the impact of ADHD on the educational outcomes of university students is presented.

**Results:**

A consensus was reached that offers expert advice, practical guidance, and recommendations to support the medical, education, and disability practitioners working with university students with ADHD.

**Conclusions:**

Practical advice, guidance, and recommendations based on expert consensus can inform the identification of ADHD in university students, personalised interventions, and educational support, as well as contribute to existing research in this topic area. There is a need to move away from prevailing notions within higher education about ADHD being a specific learning difference (or difficulty) and attend to the urgent need for university students with ADHD to have timely access to treatment and support. A multimodal approach can be adapted to support university students with ADHD. This approach would view timely access to treatment, including reasonable adjustments and educational support, as having a positive impact on the academic performance and achievement of university students with ADHD.

## Background

Going to university can be an exciting experience, but it is also a daunting and stressful experience for new and returning students. The pressure to do well academically and cope with an array of lifestyle changes, can impact on the mental health and wellbeing of university students, especially students with ADHD who are transitioning from adolescence into adulthood [1]. This transitional phase defines a critical developmental stage in life termed “emerging adulthood” [2]. Institutions of higher education (HEIs or universities) are arguably designed for the kind of identity exploration that defines emerging adulthood. This includes leaving home to go to university, and perhaps for the first time, being independent and responsible for managing one’s own finances and dietary needs, whilst at the same time being exposed to a multitude of different worldviews and new opportunities for friendships, romances, partying and work [3]. Emerging adulthood is also recognised as a peak period for experimentation with substance use or high-risk sexual and other behaviours, and for the onset or exacerbation of mental health problems including self-harm and suicide [4]. The mental health and wellbeing of university students is a cause for concern [1, 5], and the experience of the expert group is that emerging adults with ADHD may be particularly vulnerable during and after transitioning to university.

ADHD is a neurodevelopmental disorder that begins in childhood and frequently persists into adulthood. ADHD is clinically defined by persisting symptoms of inattention, hyperactivity and impulsivity that can cause functional impairments in multiple domains of daily life. In the Diagnostic and Statistical Manual version 5 (DSM-5) [6], and the International Classification of Diseases version 11 (ICD-11) [7], diagnostic requirements for ADHD are broadly similar. For this reason, and since the ICD-11 officially comes into effect in January 2022, in this report, reference is made to DSM-5 diagnostic requirements for ADHD in adults. Table 1 lists some typical characteristics and behaviours seen in adults with ADHD, including university students. It is also not uncommon for university students with ADHD to present with co-occurring specific learning differences (or difficulties) (SpLDs), developmental co-ordination disorder (DCD) or dyspraxia as the former term, autism spectrum disorder (ASD), anxiety, depression, personality, eating, and substance use disorders [8–14]. A significant majority of university students with ADHD will experience academic difficulties to varying degrees of severity [15, 16]. Previous studies refer to “educational or academic outcomes” in terms of academic achievement (*attainment of information and skills learnt, grades obtained on continuous assessments such as standardised examinations or coursework*) and academic performance (*completed years of schooling, enrolment into university, final grades awarded, retention, and progression*) [17]. Evidence suggests ADHD will impact on these different academic domains in a negative way [18].

**Table 1:** Typical characteristics of ADHD and associated behaviours in adults with ADHD Adapted from Asherson et al., [19], Nigg [20], Sedgwick [21]

Typical characteristics	Associated behaviours
Inattention (“attention deficit”)	Quickly losing focus, shifting attention, absent-mindedness.
	Easily distracted by low-priority activities or activities that other people tend to ignore.
	Spontaneous mind-wandering, “zoning out” or daydreaming which makes it hard to focus on reading, writing, or listening to others.
	Can overlook details, which leads to errors or incomplete work.
	Quietly getting bored, especially when the novelty has worn off.
Hyper-attentiveness (or hyper-focus – a paradoxical symptom)	May be a coping mechanism for distraction – tuning out and becoming totally absorbed in self interesting, stimulating or rewarding tasks and activities.
Disorganisation & forgetfulness	Poor organizational skills (e.g., problems with planning, goal setting, decision making, keeping track of tasks/responsibilities, problem solving).
	Procrastination, poor time management, forgetting commitments, appointments, or deadlines.
	A habit of losing or misplacing things (keys, wallet, phone, documents).
Hyperactivity, restlessness, fidgety or having lots of energy	Feeling agitated, inner restlessness, always “on the go” as if driven by a motor, talking excessively, trouble sitting still, or tapping.
	Getting quickly or easily bored, craving excitement or stimulation.
	Engaging in high intensity or extreme sports/activities that involve speed, height, a high level of physical exertion and highly specialised gear.
Impulsivity	Interrupting others or talking over them; blurting out thoughts or saying things without thinking.
	Engaging in reckless or risk behaviours without much concern for the consequences (e.g., spontaneous sexual “hook-ups”, gambling, Internet overuse, binge drinking or drug taking, driving too fast).
Emotional lability (or emotional dysregulation)	Regular feelings of irritability, inability to cope, short or explosive temper, being easily flustered and/or stressed, hypersensitive to criticism.
	Low self-esteem, sense of underachievement, constantly worrying about making the same mistakes, not meeting obligations, fatigue or burn-out, finding it hard to stay motivated.

### Historical context

The historical context matters a lot for understanding the ways in which ADHD exists in society, including how it is perceived, experienced, and managed. Within UK HEIs, ADHD is perceived and/or conceptualised as a SpLD [22]. In the special educational needs and disability (SEND) code of practice (0 to 25 years), ADHD is conceptualised as a social, emotional, and mental health difficulty [23], and in the DSM-5 and ICD-11, ADHD is defined as “*the most common mental health disorder in childhood that often persists in adulthood*” [6, 7]. These conceptual differences reflect how the nomenclature, understanding of functional impairments, and clinical characteristics of ADHD within different professional contexts have evolved over time. However for some authors, it was the inception of compulsory education in the late nineteenth century, rather than advances in the medical sciences, that transformed ADHD into a salient societal concern [24]. In the UK, when compulsory education was first instituted, government funding to schools including salaries for teachers, was based on the numbers of students that attended school for at least 100 days per academic year and passed standardised examinations in the 3Rs (reading, writing, arithmetic) [25]. This system, known at the time as “payment by results” [26], is said to have also motivated teachers to raise concerns about students who struggled to pass the 3Rs examinations, and eventually these students were deemed uneducable in mainstream schools [27, 28]. Some of these students were described as “… *hyperactive, distractible, unruly and unmanageable in school … frequently disturbing the whole class … quarrelsome and impulsive … often leaving the school building during class time without permission*” [29], p.15).

The Egerton Royal Commission [30], was first to examine the problem of uneducable students in mainstream schools. In its final report the umbrella term “feeble-minded”, although pejorative today, was introduced to categorise students assessed and certified as needing special education. Arguably, feeble-mindedness is the antecedent for a variety of social, emotional, mental and physical health difficulties that can cause learning problems for a sub-set of students. The early use of the term in education also marked the medicalisation of poor scholastic performance and failure [31]. Although Still’s observation of a “*moral defect without intellectual impairment*” in school children [32], was heralded as an early descriptor of the contemporary medical concept of ADHD [33], the term feeble-minded categorised all “*children who could not be properly taught in ordinary elementary schools by ordinary methods,*” and this included the children who Still had described [34]. In the early twentieth century, new research on the heritability of intelligence roused a relentless eugenic enterprise to eradicate feeble-mindedness by preventing its procreation [35]. These events coincided with the development of psychometric tests of intelligence [36–38], and their use within education became the means by which students were differentiated as either feeble-minded or “simply dull/backward”. The former group of students were sent to newly established residential colonies for care and management under the Mental Deficiency Act 1913, whilst the dull/backward students continued to be educated within mainstream schools [39].

In 1913, Cyril Burt (1883–1971), the father of educational psychology in the UK, was the first psychologist to be appointed by the London County Council (LCC) to assess students referred under the Mental Deficiency Act. Burt administered psychometric tests with these students, conducted extensive ground-breaking research into educational backwardness, developed standardised tests for use in schools and provided teachers with psychological advice on how best to manage emotional and behavioural disorders in students [40]. Through his work, Burt argued that intellectual ability was on a continuum, intelligence between boys and girls was the same, academic performance and achievement was variable, and that learning differences (or difficulties) observed in students considered dull, backward, feeble-minded or maladjusted, constituted a single problem [41–44]. Burt’s seminal work on educational backwardness was insightful, in the sense that it not only associated causes of backwardness in students with low scores on a psychometric test or other environmental factors, but also with disorders of temperament and conduct. One category within these disorders was the “excitable and unrepressed child” [44], and descriptors of this disorder are clearly akin to the characteristics of ADHD known today. Interestingly, Burt published his work on the “backward child” in 1937, the same year that Charles Bradley in the USA reported on the positive effects of psychostimulant medication in students who exhibited various behaviour disorders [45].

The influence of Burt’s work on educational policy and provisions for students with special educational needs was profound [46]. It was reflected in the landmark Warnock Report on special education [47]. The recommendations of Warnock Report compelled legislators to enshrine the policy of inclusion within the Education Act 1981, and to introduce the broad concept of “special educational needs” (SEN) to categorise students with a range of learning difficulties and/or disabilities. Descriptors of SEN have since transformed into those listed in the current SEND code of practice (0 to 25 years) [23]. But despite all this early work, ADHD has continued to be a contentious and controversial medical diagnosis in UK, with one study reporting that only “*73 hyperactive children were seen at the Maudsley and Bethlem Royal Hospital in London between 1968 and 1980*” [48], p.16–17). Following the publication of a protocol for the treatment of ADHD based on DSM-IV criteria [49], diagnostic rates of ADHD increased in the UK and continued to do so with subsequent publications of clinical guidance for the diagnosis and management of ADHD in children, young people, and adults [50]. There are still many challenges with regards to timely access to diagnosis and treatment for university students with ADHD, and support for practitioners and educators who have reported ADHD as one of the most challenging disorders to deal with in university students [51]. These views echoed in the Institute for Employment Studies (IES) report on support for disabled students in higher education in England for the Office for Students (OfS) [52]. This IES report noted that “… *providers* [university disability services] *were facing a number of, often shared, challenges* ...” (p.132), which included dealing with a rising numbers of university students with ADHD and complex mental health needs. One provider quoted by the IES said that:

*“… the support provisions for disabled students is understandably being affected by external factors. How to manage that impact is a focus for the disability and dyslexia team… this includes… the number of students with ADHD which has grown dramatically in recent years. This group of students are very challenging to support for both the service and for academic staff. The disability and dyslexia service need training and development to enable them to both support these students and the academic staff working with them ...*” [52] p.134).

### Effects of ADHD within higher education

In the UK, across Europe and worldwide, there is a paucity of research about university students with ADHD. Previous studies mostly seem to originate from North America, where research activity in this topic area has been ongoing since the 1990s, and the impact of ADHD on the educational outcomes of college (or university) students is more widely understood. A comprehensive review of these studies was conducted by Sedgwick [21], and a summary of the main findings are presented in Table 2.

**Table 2:** University students with ADHD – Summary of key findings (From Sedgwick [21]

Theme	Findings
**Academic, social & psychological functioning**	● Poor performance in time-limited exams and poor overall academic achievement. ● Lower levels of social adjustment, social skills, and self-esteem in relationships. ● A range of factors predicted academic success including better coping strategies, a positive mental attitude/resilience and physical exercise.
**Giftedness**	● High intelligence quotient (IQ) does not preclude the possibility of having ADHD. ● Students who get good grades but still report ADHD related symptoms are most at risk of not getting diagnosed and treated.
**New media technologies (NMTech)**	● NMTech could precipitate or perpetuate ADHD-related behaviours. ● Internet overuse (or addiction). ● Important to ask about NMTech use during assessments for ADHD.
**Treatment**	● Paucity of research in university students with ADHD. ● Academic performance and achievement improve with medical treatment. ● Coaching is not defined as a psychological treatment, but it may be useful.
**Substance misuse & non-medical use of stimulants**	● Likely to misuse tobacco, alcohol and other licit or illicit substances. ● Prevalence rates for use of psychostimulants as “study drugs” is between 5 and 35% in North American and 0.8–16% in Europe, but even lower in Ireland and the UK.
**Malingering**	● Concerns about students feigning ADHD to get a prescription for stimulant medication, but detection depends on the knowledge, skills and expertise of the practitioner undertaking a diagnostic assessment.

#### ADHD and intellectual giftedness

The relevance of intellectual giftedness to university students with ADHD was considered by the expert group. Intellectual giftedness is another contested concept variously defined as exceptional intellectual ability, academic talent, or high-potential learners, with concurrent traits of creativity, curiosity, effort, and self-motivation [53–56]. Intellectual giftedness is referenced in the Canadian ADHD Practice Guidelines [57], but not in the DSM-5 or ICD-11 [6, 7], or other clinical guidelines [50]. Research suggests that intellectual giftedness can either over-shadow or compensate for attention difficulties, or the behaviours associated with ADHD can over-shadow traits of intellectual giftedness, and that students with both ADHD and intellectual giftedness can be difficult to identify or assess using standardised measures and observational checklists [58–62]. The co-existence of ADHD in intellectually gifted individuals, including university students, is controversial. The theories of positive disintegration [63], and asynchronous development [64], have both been used to understand various aspects of intellectual giftedness in students with ADHD. Important areas of current research include the potential misdiagnosis of intellectual giftedness as ADHD, and the occurrence of ADHD and intellectual giftedness as a dual diagnosis [65].

Intellectual giftedness in students with ADHD is thought to be under-identified by parents, educators, psychologists, and physicians. Brown et al., for instance, reported that “*adults with IQ scores in and above superior range have often sought evaluation and treatment for chronic difficulties with organizing their work, excessive procrastination, inconsistent effort, excessive forgetfulness, and lack of adequate focus for school and/or employment. They question whether they might have an attention deficit disorder, but often they have been told by educators and clinicians that their superior intelligence precludes having ADHD*” [66], p.161).

Intellectual giftedness does not preclude having ADHD, and in some university students with ADHD it could mitigate some deficits in executive function and allow them to flourish academically or to go on and have successful careers [67–69]. Some authors proposed that a degree of autism (or savantism) could foster a special talent in gifted individuals [70], including individuals with ADHD [71, 72]. Other authors warn that intellectual giftedness may only be a protective factor for students with ADHD during their pre-18 school years [59, 73]. This may change when they transition into higher education where self-directed learning becomes an essential academic skill and when challenges such as living away from a structured home environment, or needing to be more organised, can precipitate a worsening of ADHD symptoms and significant levels of impairment start to emerge [74, 75]. These issues may become more apparent in post-graduate students, who are selected based on their undergraduate academic achievements [56, 76, 77]. Empirical studies between 2000 and 2014 about the identification, misdiagnosis and dual diagnosis of intellectual giftedness and ADHD were reviewed by Mullet and Rinn, [65]. From this review, traits of intellectual giftedness versus ADHD have been compiled for the purposes of clarity. These are listed in Table 3 below.

**Table 3:** Differentiating Giftedness and ADHD Compiled from Mullet and Rinn [65]

Indicators of Intellectual giftedness	Indicators of ADHD
Boredom, daydreaming and attentional difficulties in unchallenging learning environments.	Boredom, daydreaming and attentional difficulties in multiple domains.
Low tolerance for tasks or activities that seem irrelevant.	Low tolerance for tasks or activities that seem irrelevant.
Extended periods of time spent on topics of self-interest.	Extended periods of time spent on topics of self-interest.
Discrepancies between intellectual, physical, emotional, and social development.	Discrepancies between intellectual, physical, emotional. and social development.
Emotional intensity may lead to power struggles with authorities.	Emotional intensity may lead to power struggles with authorities.
Questions or challenges rules, regulations, customs, and traditions.	Poor impulsive control may cause difficulties adhering to rules, regulations, customs, and traditions.
High activity levels that is focused and directed, restlessness, may need less sleep.	High activity levels that is random rather than goal directed, restlessness, problems with sleep.
Tendency to answer correctly when responds impulsively to questions.	Tendency to guess incorrect answers when responds impulsively to questions.
Prone to social problems and struggles in social relationships.	Difficulties regulating or inhibiting behaviour in social contexts, prone to social problems and struggles in social relationships.

In sum, this report presents a selective review of previously published literature on ADHD in university students and consensus based on expert opinions. It aims to critically examine and discuss the impact of ADHD on educational outcomes of university students and provide evidence-based, practical advice and guidance on how best to support these students during their programme of studies. Expert consensual advice and guidance in relation to screening and diagnostic assessments for ADHD in adults, specific interventions for university students with ADHD, a potential model for service provision, staff training and development, will contribute to existing research in this topic area.

## Method

The purpose of the expert consensus meeting was to formulate practical advice, guidance, and recommendations for supporting medical, mental health, educational and disability practitioners who work with university students with ADHD. This report is based on previously published literature that was identified, selected, collated, and critically reviewed using a framework for scoping studies [78], as well as the professional experience of the expert group. The consensus meeting was convened by the UK Adult ADHD Network (www.UKAAN.org) in July 2017. UKAAN is an organisation founded in 2009 by a group of mental health specialists, responding to NICE guidelines [50], and recommendations from the British Association for Psychopharmacology (BAP) [79, 80], for the purpose of providing support, research, education, and training to professionals working with adults with ADHD. The aims of the consensus meeting were to address the following questions:

1. Is ADHD a hidden disability within higher education institutions (HEIs)?
2. Is ADHD a specific learning (difficulty) or difference?
  *What are the similarities and differences between ADHD, specific learning (difficulties) or differences & other mental health conditions?*
  *What is the impact of stigma?*
3. What constitutes best practice for supporting university students with ADHD?
  *Service provision*
  *Screening & diagnostic testing*
  *Pharmacological & non-pharmacological interventions*
  *Staff training and development*

Meeting attendees included the authors and 48 other mental health, neurodiversity, and disability practitioners, learning assessors and 2 university students with ADHD from England, Wales, and Scotland. The authors who attended the meeting represented a multidisciplinary group of prescribing and non-prescribing clinicians, practitioners, and academics, with extensive experience and expertise in working with adults with ADHD, including university students. Attendees engaged in conversations throughout the day with the aim of achieving consensus. The meeting was structured around presentations on relevant topics that are listed below, and the personal accounts from the 2 university students with ADHD, followed by questions, and answers (Q&As).

The first author facilitated discussions among the attendees to elicit verbal accounts of experience and to reach a consensus position on the topic being discussed. At the end of the meeting, the first author presented a summary of the main points previously agreed (which are listed in Table 4), and then asked the attendees to raise a hand to indicate whether they agreed with each point being raised. This is line with the phenomenological methodological framework that was used to gain an emic or “insiders” perspective of the attendee’s experiences, knowledge, and expertise of working with university students with ADHD [81, 82]. The consensus meeting started with an overview of the neurobiology of ADHD to set the scene, then invited speakers presented on the following topics:

- The effectiveness of stimulant medication in treating ADHD.
- Academic coaching for university students with ADHD.
- The SpLD Assessment Standards Committee (SASC) guidelines for the assessment of ADHD in university students.
- Tele-psychiatry: Internet based treatment services for university student with ADHD.
- The student experience: What is it like to be a university student with ADHD?

**Table 4:** Summary of the main consensus points

i. ADHD is a hidden disability and should no longer be categorised as a specific learning difference/ difficulty (SpLD) in higher education.	
ii. There is a need to overcome the stigma associated with having ADHD.	
iii. There is a lack of access to assessment and treatment for university students with ADHD. Many of these students are assessed by a specialist teacher assessor or educational psychologist and get a recommendation for reasonable adjustments and a referral to their GP to access specialist medical treatment.	
iv. There are long waiting lists to be seen by NHS specialist adult ADHD clinics.	
v. There is a need to develop rapid access care pathways for the medical treatment of ADHD in university students.	
vi. Some students with ADHD do not perform well at university, whereas other students performed very well, and what seemed to make the difference was the level of personalised support that they received.	
vii. There is a need to develop training that includes psychoeducation, how to screen for (and diagnostically assess) ADHD and use recommended strategies for supporting students with ADHD in higher education.	
viii. Best practice for supporting university students with ADHD would entail joint/ collaborative working between university disability services and NHS or private service providers.	

The attendees and speakers consented to the presentations and discussions being audio recorded. After the meeting, the recording was transcribed verbatim with care taken to remove all identifiable information. Authorship of the manuscript was based on involvement during the meeting, a willingness to work on the manuscript after the meeting, clinical and professional expertise in the assessment and treatment of ADHD in university students. The first author (JSM) consolidated the presentations, data from the transcripts and notes relevant to the main points agreed in the meeting, into a manuscript that was circulated amongst the authors for review, revision, final agreement, and approval. This manuscript reflects the clinical experience and expertise of the authors and is supported by published literature.

## Results and consensus outcome

The series of questions and summary of main points addressed during the meeting were collated and are discussed below. A summary of the main recommendations is listed in Table 10.

### Is ADHD a hidden disability within higher education institutions (HEIs)?

Only one study was found that reported on the prevalence of ADHD in UK university students. In this study Pope et al. [83] used the Conners’ Adult ADHD Self-Rating Scale to assess for symptoms of ADHD in 1185 undergraduate psychology students from four UK universities. The findings revealed that about 7% of these students self-reported above-threshold symptoms of ADHD. In a study from the USA, DuPaul et al. [84] reported that at least 25% of college students with disabilities were diagnosed with ADHD. Among university students in China (*n* = 343), and in the USA (*n* = 283), ADHD was reported to be around 5% in the USA cohort and 8% in the Chinese cohort [85]. These data clearly depict variability, with some reported rates suggesting a higher prevalence of ADHD among university students, when compared to the reported worldwide prevalence estimate of 2–3% for ADHD in adults [10]. However the studies that reported higher prevalence estimates (e.g., Norvilitis et al. [85] did seem to have determined the presence of ADHD based on a count of symptoms alone, and did not assess functional impairments to meet full diagnostic requirements for ADHD. Perhaps if functional impairments had also been considered, prevalence rates of ADHD in university students may have been similar to the prevalence rates reported for adults [86].

**Fig. 1:**
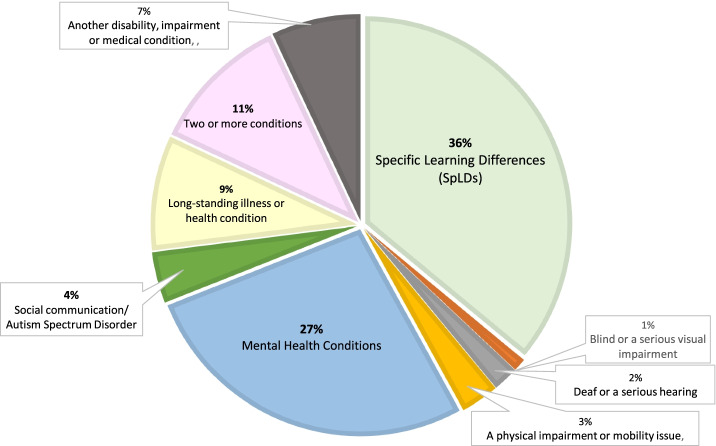
Disabled university students in the UK. Source: Table 15: *UK-domiciled student enrolments by disability and sex*, for the academic year 2018-19, (total number of disabled students 316,380) [22]. NB: There are high rates of overlap between ADHD and both SpLDs and mental health conditions, but the prevalence of ADHD is unknown, because there is no separate category for it

University students with ADHD are part of a much bigger group of disabled students that are represented within the *widening participation (WP) strategy* that forms a major component of higher education policy in the UK [87]. The WP strategy requires HEIs in the UK to collect, analyse, and respond to data on disabled students. To do so, HEIs utilise UCAS (Universities and Colleges Admissions Service), codes and categories of disability listed in Table 5. As shown, ADHD is listed “*G – Specific Learning Difference e.g., dyslexia, dyspraxia, or ADHD*.” The Higher Education Statistical Agency (HESA, https://www.hesa.ac.uk) also collect, process, and publish data about disabled students within higher education in the UK. Figure 1 depicts percentages of the HESA Data for “*UK domiciled students’ enrolments by disability and sex”* based on a total number of 307,975 for the academic years 2014/15–2018/19 [22]. From this data it is also not possible to ascertain a prevalence estimate for ADHD among university students or even to identify if ADHD exists within higher education.

**Table 5:** Current UCAS codes/categories and definitions of disability

**A**	No Disability
**B**	Social/communication impairment such as Asperger’s Syndrome/other Autistic Spectrum Disorder
**C**	Blind/serious visual impairment uncorrected by glasses
**D**	Deaf/serious hearing impairment
**E**	Long standing illness or health condition such as cancer, HIV, diabetes, chronic heart disease, or epilepsy
**F**	Mental Health Condition, such as depression, schizophrenia, or anxiety disorder
**G**	Specific Learning Difference e.g., dyslexia, dyspraxia, or ADHD
**H**	Physical Impairment or mobility issues
**I**	Disability, impairment, or medical condition not listed above
**J**	You have two or more impairments and/or disabling medical conditions

**Fig. 2:**
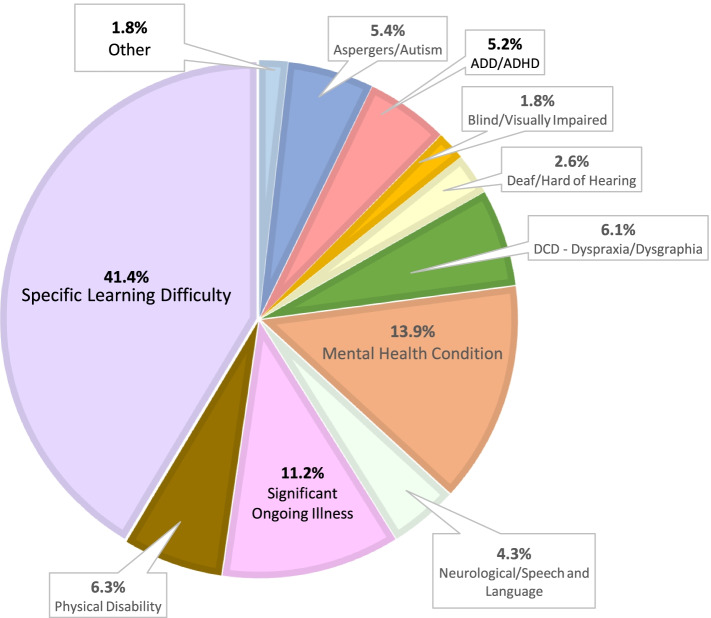
Disabled university students in Ireland. Source: Fig. 3 Breakdown of students by Category of Disability 2016/17 (total number of disabled students 12,630) [88]

Figure 2 depicts in percentages published data from 25 HEIs in Ireland, based on a total number of 12,630 university students who declared a disability for the academic year 2016/17 [88]. There are clear similarities between this data and the HESA data depicted in Fig. 1. But there are also differences in the numbers of university students who declared a mental health condition (27% in the UK vs. 13.9% in Ireland), a specific learning difference (UK 36% vs. Ireland 41.4%) and autism spectrum disorder/ASD (UK 4% vs. Ireland 5.4%). In Ireland, data is also collected on university students who declare a developmental co-ordination disorder (DCD, or dyspraxia, 6.1%) and ADHD (5.2%), but similar data is not collected in the UK. During the consensus meeting there was unanimous agreement that ADHD should no longer be subsumed under the category of a SpLD. The obvious consequence of continuing to do so is that a prevalence estimate for ADHD in UK university students will always be hard to ascertain.

### Recommendation 1. The categorisation of ADHD

The expert group recommends that ADHD should no longer be subsumed under the category of a SpLD in HESA data return categories or by university services and should be coded or categorised separately. If ADHD continues to be coded or categorised as an SpLD then no specific data about the numbers of university students who declare ADHD as a disability within UK HEIs will be collected. ADHD is a mental health condition and not a SpLD. ADHD has specific diagnostic criteria within the DSM-5 [6], and ICD-11 [7], as well as efficacious treatments (medication and psychosocial interventions) [89, 90]. A separate code to categorise ADHD within UK HEIs could result in greater recognition of the disorder and increase understanding about how it impacts on academic performance and achievement.

### What are the differences between ADHD and SpLDs?

Dyslexia, dyscalculia, dysgraphia, and dyspraxia (or DCD) and ADHD are all categorised as SpLDs within UK HEIs. However, in the DSM-5, dyslexia, dyscalculia, and dysgraphia are grouped together under a single diagnostic category of “specific learning disorder” (SLD, or learning disorder), whilst DCD is classified separately as a motor disorder and ADHD as a neurodevelopmental disorder [6]. SpLDs are not synonymous with SLD, but a university student who has been diagnosed with a SLD can also expect to meet criteria for a SpLD, be registered as disabled and qualify for reasonable adjustments under the Equality Act 2010. Specifiers and characteristics of SLD and typical SpLD terms used in higher education are listed in Table 6. Unlike ADHD, there are no known medical treatments for SLD (or SpLDs), therefore reasonable adjustments (or accommodations) are required to limit their impact within educational settings. Reading disorder (RD, e.g., dyslexia) is the most prevalent SpLD reported to account for up to 80% of all SpLDs [91]. Bidirectional comorbidity between RD and ADHD which is estimated at 25–40%, is likely due to shared genetic risk factors [92]. This may also explain why deficits in executive function are seen in both ADHD and RD [93, 94]. Executive functions (EF) are described as a set of top-down mental skills essential for academic performance. In Table 7, EFs are conceptualised in terms of their organisational and regulatory functions. The three commonly described EFs are inhibitory control, working memory and cognitive flexibility [95, 96]. Although research suggests that deficits in EF can adversely impact academic functioning due to the problems they can cause with sustaining attention, forgetfulness, procrastination, organisation skills, prioritising, regulating alertness, emotional and behavioural self-control, psychometric tests of EF are still not sensitive enough to assess for the core deficits of ADHD [97–103].

**Table 6:** Specifiers and Characteristics of SLD [6], and typical SpLD terms [104–106]

SLD with impairment in	Characteristics	Typical SpLDs
Reading (reading disorder, RD)	Deficits in *decoding* speech sounds of words (phonological weaknesses) and *fluency* (not reading accurately with adequate speed)	Dyslexia
Mathematics (maths disorder, MD)	Deficits in number sense, memorisation of arithmetic facts, accurate or fluent calculation or math reasoning	Dyscalculia
Written expression (writing disorder, WD)	Deficits in *orthographic coding* (e.g. spelling, grammar, punctuation, capitalisation) and *finger sequencing* (the movement of muscles needed for writing)	Dysgraphia

**Table 7:** Examples of organisation and regulatory roles of executive functions [95]

ORGANISATION (gathering information & structuring it for evaluation)	REGULATION (Evaluating available information & modulating environmental responses)
Language/ rule acquisition	Initiating & inhibiting context specific action/ behaviour
Attention, staying focused	Motivation
Planning	Self-control, self-monitoring
Sequencing, prioritising	Moral reasoning
Problem-solving (fluid intelligence)	Emotional regulation
Thinking about 2 or more concepts simultaneously	Decision-making
Abstract thinking	
Selecting/ filtering relevant sensory information	

**Fig. 3:**
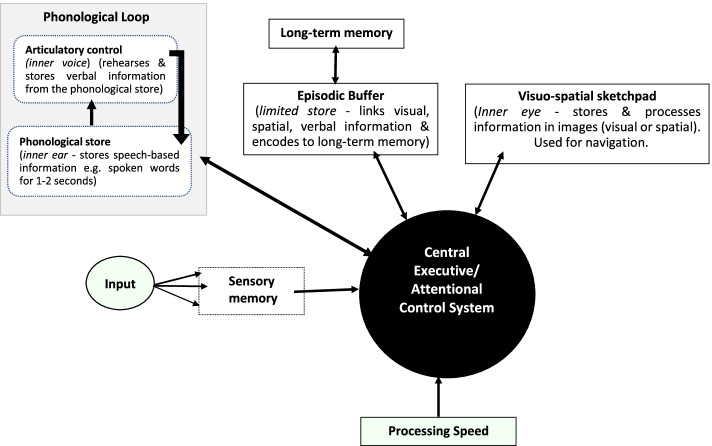
Model of Working Memory (Adapted from Baddeley [111]

The Baddeley and Hitch [107] conceptual model of working memory (WM) in Fig. 3, proposes that WM is a core EF for storing and manipulating information, and with a central role in attention, allocating data to its slave systems (phonological loop and visuo-spatial sketchpad), performing task switching, mental arithmetic, problem solving and interfacing with long-term memory through the episodic buffer. The episodic buffer acts as a temporary store for the phonological loop, which processes spoken and written information, whilst the visuo-spatial sketchpad processes visual imagery. Although this model can be used to understand the importance of WM in academic tasks such as reading, comprehension, verbal reasoning (phonological loop), navigation (visuo-spatial processing) and problem-solving (central executive) [107–111], the model can also be used to understand how deficits in WM might occur in both ADHD and reading disorder [93]. Reading disorder (e.g., dyslexia) is defined by deficits in *decoding* the speech sounds of words and structure of language (phonological weakness), *fluency* (an inability to ready quickly with appropriate expression) and processing speed [11, 91, 93, 102].

Processing speed (PS) is not an EF per se, rather it is said to be a cognitive ability that describes the amount of time it takes to identify, understand, react, or respond to information received, whether it be visual (letters and numbers), auditory (language) or movement [112]. Since PS is surmised to impact on WM, phonological loop and visuo-spatial sketchpad processes, and the fine motor co-ordination associated with DCD, it’s impact on academic performance is also said to be direct [113]. PS is an index score on the WAIS (Wechsler Adult Intelligence Scale), measured by rapid automatized naming of pictured objects, letters, numbers, and colours [112]. Slow PS or PS deficits, often identified by a low PS score on the WAIS, has been associated with reading disorder [102], ASD and ADHD [114]. This also means when a student is identified with PS deficits on the WAIS for instance, certain academic tasks, such as an examination which requires “*an ability to quickly come up with an answer and retrieve information from memory*”, may take longer to complete, hence these students tend to be awarded extra writing time for examinations as a reasonable adjustment [115], p4). PS deficits are also implicated in the comorbidity between ADHD and reading disorder [116], the combined effect of which may produce more severe learning problems than when each of these disorders occurs on its own [11, 117, 118]. High rates of comorbidity are also reported between ADHD and other SpLDs (e.g., dyscalculia and dysgraphia), and other disorders such as DCD and ASD, with similar combined effects as those surmised between ADHD and RD, but a paucity of research limits understanding of the severity of cognitive deficits in these comorbidities and their impact on academic functioning [8, 14, 70, 119–121].

### Recommendation 2. ADHD and SpLDs

Comorbidity between ADHD and other neurodevelopment disorders, which include SpLDs, adversely impacts on academic functioning. The expert group therefore recommends screening for ADHD as part of routine practice for university students who report learning difficulties that seem to be associated with dyslexia, dyscalculia, dysgraphia, dyspraxia and/or ASD, not only because these conditions are highly likely to co-occur [8, 11, 14], but ADHD can be missed if a student is only screened for SpLDs and/or ASD. For students that screen positive for ADHD, a referral for treatment and management by a suitably qualified mental health professional (e.g., student health GP, psychiatrist, or mental health nurse/practitioner) is important. Although ADHD on its own can provide an explanation for learning problems within higher education, it can also add complexity to the learning problems associated with SpLDs, DCD or ASD. These complexities need to be considered when assessing for, and/or awarding reasonable adjustments. Screening tools that are used in routine practice are listed in Table 8.

**Table 8:** Examples of screening questionnaire & diagnostic tools

**Screening tools**	**Uses**
Adult ADHD Self-Report Scale (ASRS) (Kessler et al., [122]. Free at: https://www.hcp.med.harvard.edu/ncs/ftpdir/adhd/ASRS-5_English.pdf ASRS-5 screener at https://www.hcp.med.harvard.edu/ncs/ftpdir/adhd/ASRS-5_English.pdf) (accessed 7/2021).	ADHD
Autism-Spectrum Quotient (AQ-10) (Allison et al., [123]. Free at: http://docs.autismresearchcentre.com/tests/AQ10.pdf (accessed 7/2021).	ASD
The Adult Dyslexia Checklist, British Dyslexia Association [124]. Free at: https://cdn.bdadyslexia.org.uk/uploads/documents/Dyslexia/Adult-Checklist-1.pdf?v=1554931003. (accessed 07/2021).	Dyslexia
Kessler Psychological Distress Scale (K10) (Kessler et al., [125].	Anxiety & depression
Penn State Worry Questionnaire (PSWQ) (Meyer et al., [126].	Pathologic worry
The Simple Screening Instrument for Substance Abuse (SSI-SA) (Center for Substance Abuse Treatment [127].	Substance misuse
The Improving Access to Psychological Therapies Manual [128], Appendices and helpful resources, at: https://www.england.nhs.uk/publication/the-improving-access-to-psychological-therapies-manual/ (accessed 7/2021). Useful tools in appendices section: • Page 33: Generalised Anxiety Disorder Scale (GAD-2) • Page 36: Generalised Anxiety Disorder Scale (GAD-7) • Page 35: Patient Health Questionnaire (PHQ-9) • Page 42: Panic Disorder Severity Scale	Anxiety, depression & panic disorder
These and other online tools are also available at: https://psychology-tools.com/	Various
**Diagnostic tools**	**Uses**
DIVA Foundation [129], The Diagnostic Interview for ADHD in Adults (DIVA-5) at: https://www.divacenter.eu/DIVA.aspx?id=523	ADHD
ACE+ [130], at: https://www.psychology-services.uk.com/adhd	ADHD
Weiss Functional Impairment Rating Scale–Self Report (WFRIS-S) [131]. Free at: https://www.caddra.ca/wp-content/uploads/WFIRS-S.pdf (accessed 7/2021).	Functional impairment

### What are the differences between ADHD and other mental health conditions?

It is equally important to differentiate ADHD from other mental health conditions and to consider the impact of these conditions on university students with ADHD when they do co-occur. Year-on-year increases in the number of students declaring a mental health condition at university have been observed, with current prevalence estimates of 27% amongst university students who declare a mental health disability before or during their programme of studies (see Fig. 1). A study by Anastopoulos et al. [16] examined rates and patterns of co-occurring disorders in 443 university students with ADHD. The findings of this study revealed that 55% of these students had at least one comorbidity whilst 32% had two or more, and that commonly reported comorbidities with ADHD were depressive and anxiety disorders. These elevated rates differ from rates reported in an epidemiological study conducted in 20 high, medium, and low-income countries involving 26,774 adults with ADHD. This study found that 23% of these adults with ADHD had at least one mental health comorbidity, while 14% had two or three comorbidities, and that commonly reported comorbidities with ADHD were also anxiety disorders (34%), mood disorders (22%), as well as behavioural disorders (15%) and substance use disorders (11%) [10]. Similar findings were reported in qualitative studies, although the participants in these studies, also reported positive aspects of ADHD such as high levels of energy and drive, creativity, hyper-focus, agreeableness, empathy, self-acceptance, and a willingness to assist others [132, 133].

During the consensus meeting the discussion mostly focused on university students who frequently reported anxiety and depression. Different types of anxiety (e.g., *generalised anxiety disorder, social anxiety, specific phobias, agoraphobia, panic disorder, substance/medication induced anxiety*), or depressive disorders (e.g., *mood dysregulation disorder, major depressive disorder, dysthymia, premenstrual dysphoria, substance/medication induced depression*), were discussed in relation to ADHD. Major depressive disorder (MDD) does show some overlap with ADHD symptoms such as poor concentration and working memory performance, but in MDD these characteristics are episodic and only arise during periods of low mood, anhedonia (loss of interest/enjoyment in ordinary experiences), or when there are ruminations dominated by negative content, and appetite disturbances, which are not characteristic of ADHD [134]. In contrast, people with ADHD usually present with attention regulation problems. This means they may be able to focus during highly stimulating or interesting tasks and activities, but problems with concentration will remain regardless of mood state [19]. Poor concentration and restlessness are also symptoms that are shared between anxiety disorders and ADHD. Anxiety disorders are characterised by fluctuations in pathologic worry, fear, and somatic symptoms, which drive concentration problems, whereas in ADHD, problems with attention and restlessness, drive concentration problems and reflect persistent traits that are independent of anxiety [134].

University students with ADHD can present to medical, counselling, and disability services with problems related to anxiety and/or depression, because challenges of university life can also play an important role in affected mental health. Both anxiety and depression are frequently co-occurring conditions in adults with ADHD [10], as well as in university students with ADHD [16]. However, it is still important to be aware that symptoms of ADHD can mimic both anxiety and depression [19], and that anxiety and depression can in turn affect attention, concentration, processing speed, and motivation, giving rise to poor performance on reading, writing, attending classes and group work [135]. University students with ADHD can also be prone to “test anxiety” and experience disabling levels of worry, emotional and somatic symptoms, that exacerbates their ability to focus and perform during evaluative assessments such as examinations. This may further increase the risk that they achieve poor grades, or delay completing their programme of studies [136, 137]. More generally, symptoms of ADHD can be misdiagnosed for anxiety, mood, or personality disorders. This may be an issue for females with ADHD whose symptoms are more likely to reflect internalising symptoms and emotional dysregulation [138].

Emotional dysregulation is a prominent feature in ADHD and is listed in the DSM-5 as a characteristic that supports the diagnosis of ADHD [6]. Research suggests that up to 80% or more adults with ADHD report significant levels of emotional dysregulation/lability marked by irritability, volatility, a hot temper, low frustration tolerance and sensitivity to criticism [139–141]. These attributes do reflect a part of the normal range of mood symptoms for people with ADHD, but if severe, then they can also be misconstrued for MDD, bipolar disorder or a personality disorder. Emotional lability (EL) in adults with ADHD tends to manifest as short-lived emotional outbursts, or feelings of irritability, frustration, or anger that is often (but not always) in response to daily events [140]. Studies on EL in adults with ADHD also suggest that it is more closely linked to the development of low self-esteem and poor self-concept, when compared to the other core features of ADHD [140, 142]. University students with ADHD who have problems with EL are more likely to encounter additional challenges with making and maintaining academic and social relationships [143], or with participating in group work, team sports, societies, or other activities at university, especially if they frequently express anger, sadness, or anxiety when with others [144].

University students with ADHD who do not cope well with anger or sadness may also use tobacco, alcohol, cannabis, or other drugs; sex, gambling, or gaming as coping strategies [145–147]. Some students with ADHD may not be able to control their alcohol intake for instance, and binge drink often or report more drinking-induced blackouts, loss of friends or romantic partners as a result of their drinking habits [147]. In the study by Rooney et al., [148], although students with ADHD did not report higher levels of alcohol use, they did report more dangerous/hazardous use. In another study when university students with ADHD escalated their substance use, they increasingly skipped classes and reductions in their academic grades were observed [149]. Although similar problems are seen in clinical practice with other drugs of abuse such as cocaine [150], some drugs are used to control symptoms of ADHD. For example, cannabis may help reduce some ADHD related problems such as restlessness, EL and problems getting to sleep [151]. In contrast to poor mental health, emotional wellbeing is increasingly being viewed as important for enhancing a student’s motivation to learn, academic performance and interpersonal skills. Studies have shown that reducing stress, and increasing enthusiasm, contentment, joy, hope, pride, exuberance, and elatedness are linked to improvements in academic self-efficacy, interest, effort, engagement, performance, and achievement [152–156]. There are also positive aspects of ADHD that can be useful at university [133].

### Recommendation 3. ADHD and mental health conditions

The expert group recommends that university students who present with enduring anxiety and depression, and report persistent problems with learning or studying, should be screened for ADHD. ADHD can mimic these conditions, and likewise, anxiety and depression can mimic ADHD. Anxiety and depression may also reflect a normal stress response to the educational and psychosocial impairments of ADHD. Screening for ADHD should therefore be conducted in all students diagnosed with, or frequently complaining about, anxiety or depression (or other chronic mental health problems), particularly when they are taking medication and there is no or only limited improvements in their mental state. For students that screen positive for ADHD, a referral for treatment and management by a suitably qualified mental health professional (e.g., student health GP, psychiatrist, or mental health nurse/practitioner), is important.

### What is the impact of stigma on university students with ADHD?

Stigmata are the beliefs, attitudes and structures that interact at an individual, group, or institutional level, to discriminate against a person based on a perceivable social characteristic that sets them aside from others [157]. ADHD, a diagnostic label, is a perceivable social characteristic that can be stigmatised as laziness, bad behaviour, or as having “special needs” [158, 159]. There are lingering myths, misconceptions, negative stereotypes, and labels associated with ADHD [160]. Some medical professionals in the UK, Europe, and Australia, have expressed doubts about whether ADHD is real, over-emphasising the aetiological role of parenting, or questioning the role of stimulant medication in its treatment [161]. In one study a group of university students were asked to rate the likelihood of interacting with, collaborating on a group project with, getting to know, becoming friends with, living with, working with, or dating a peer with either ADHD, a general medical condition, or an ambiguous flaw such as perfectionism. Peers with ADHD were rated as less socially desirable than peers in the other two groups [162]. In young people with ADHD, although self-stigma can present as a sense of feeling different from same age peers or by negative self-evaluations, some young people have also challenged ADHD related stigma by openly disclosing and talking about their diagnosis [163].

Some professionals may fear treating a “fake disease” or causing a drug dependency by prescribing stimulant medication, even though there is no empirical evidence to support these views [50, 158, 164]. Missing or failing to identify ADHD is more likely to happen in university students who are intellectually gifted, getting good grades, or in those, particularly females, who may be misdiagnosed with anxiety, depression, eating or personality disorders [50, 138, 158]. Some studies from the USA suggest that university students without ADHD can malinger for the purposes of obtaining a prescription for stimulant medication for use as “study drugs” [165, 166]. Malingering with ADHD for this purpose may be a phenomenon more often observed in the USA, where ADHD is more commonly diagnosed and treated in primary care. This is not the same as in the UK and Europe more generally, where ADHD in adults is an under-diagnosed and under-treated condition and suitably qualified and trained medical or non-medical prescribers (e.g. mental health nurses or pharmacists) treat ADHD [19]. From the perspective of the expert group, concerns about malingering can further stigmatise university students with ADHD in the UK, as well as discourage disclosure, bias the way a screening or diagnostic assessment is conducted and result in a failure to recognise a legitimate disorder with an effective treatment. The experience of the expert group is that malingering with ADHD is not common (even unusual) for university students in the UK. Instead, they tend to work exceptionally hard to overcome their deficits associated with ADHD and still experience academic outcomes that fall below that expected from their general intellectual ability. The need to tackle the stigma associated with ADHD was discussed during the consensus meeting, in terms of how it deterred disclosure, seeking a formal diagnosis, taking medication, or seeking additional support. Concerns about disclosing ADHD (or other mental health conditions) were also noted in the Institute for Employment Studies report to the Office for Students [52].

### Recommendation 4. ADHD and stigma

The expert group recommends that targeted programmes of training for university student support staff should include psychoeducation, how to screen for ADHD and use recommended strategies for supporting university students with ADHD. This training can also be used to raise awareness about the potential stigma associated with ADHD, its consequences and potential impact on the screening and diagnostic process, willingness to disclose ADHD at university and accept treatment.

### What is best practice for supporting university students with ADHD?

In the UK, clinical guidance recommends that the medical diagnosis of ADHD must be done by a suitably qualified practitioner, and with primary care staff providing support through shared care protocols [50]. The expert group is aware that at present, waiting times for access to treatment via specialist NHS adult ADHD clinics can be anything of up to two years or longer in some areas of the country. Given the high cost of tuition fees for university and living expenses, plus added pressures to complete a university degree on time, students with ADHD simply cannot afford to wait two or more years to access treatment in specialist NHS services, without risking poor academic performance, failure, drop-out or increased burden of illness. For some of these students the misuse of caffeine products, cannabis, alcohol, or stimulants (licit or illicit) may seem like attractive options for self-medication. Seeking an educational diagnosis of a SpLD, funded through the university disability service, maybe an attractive option that can enable access to educational support. But if the core symptoms of ADHD remain untreated, students with ADHD can continue to experience learning (and possibly other) problems during their time at university.

In one systematic review of 176 studies about the long-term educational outcomes of untreated versus treated ADHD, academic outcomes were found to be worse in students with untreated ADHD when compared to their non-ADHD peers, after controlling for IQ [18]. Another finding was that academic outcomes improved significantly when multimodal treatment was used, in comparison to when pharmacological or non-pharmacological treatments were used alone [18]. The provision of rapid access to treatment for university students with ADHD maybe challenging for clinicians working in specialist NHS services. But the expert group has found that some HEIs are using funds from their disability services budget to fund private diagnostic assessments for their students, and are commissioning medical treatment (e.g., bespoke services through the NHS or privately). These HEIs in turn note these initiatives in their “access and participation plans” (APPs) for the OfS, to demonstrate how they are improving equality of opportunity for students with ADHD, who traditionally experience poor educational access, achievement, and attainment [21].

### Recommendation 5. Service provision

**Fig. 4:**
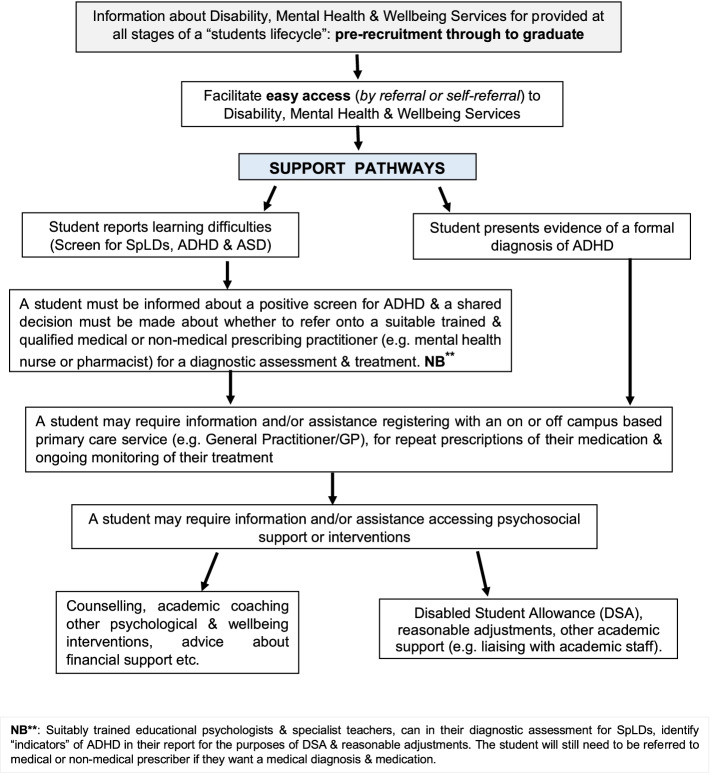
Potential Support pathway for university students with ADHD

The expert group recommends that a rapid access pathway of care for university students with ADHD be developed collaboratively between university central support services, and NHS primary and secondary care, or private providers. University disability services currently fund diagnostic assessments for SpLDs. This budget could also be made available to university students with ADHD to enable them to at least obtain a diagnostic assessment and reasonable adjustments. The expert group provides an example of a potential support pathway for university students with ADHD, which is presented in Fig. 4.

### Which screening tools and diagnostic assessments are useful?

Screening tools are used to indicate if symptoms of ADHD and/or any other co-occurring conditions that are likely to complicate the learning problems that university students with ADHD are present or not. Screening for ADHD and other potential comorbidities is done routinely in clinical practice, because it’s important to differentiate the conditions underlying the student’s presenting symptoms and consider whether they may or may not require additional reasonable adjustments or support from other services (e.g., GP, mental health, or counselling). A widely used screening tool for ADHD based on DSM-5 diagnostic criteria, is the World Health Organisation Adult ADHD Self-Report Scale (ASRSv1.1) [122], now updated to an online DSM-5 version (see Table 8 for further details and weblinks). The 18-item ASRS consists of all the diagnostic symptoms of ADHD and is useful as a screener for gathering information about ADHD symptoms that can be examined more in-depth during a diagnostic assessment. If the ASRS screener is positive for ADHD, and there are indications of sustained difficulties with attention, motor restlessness/overactivity or impulsive behaviour, then it must trigger a full diagnostic assessment by a suitably qualified practitioner.

The SpLD Assessment and Standards Committee (SASC) guidance for the assessment of ADHD, also states that “*practitioner psychologists and specialist teacher assessors who have relevant training can identify specific learning difficulties and patterns of behaviour that together would strongly suggest a student has ADHD; and in this situation they can make relevant recommendations for support at Further and or Higher Education institutions. Such diagnostic assessments should be accepted by SFE in support of an application for Disabled Students’ Allowance*” [167], p.2). This means university students can have indicators of ADHD identified as part of a SpLD diagnostic assessment and then use their diagnostic report to apply for reasonable adjustments and DSA (Disabled Student Allowance). However, even with additional educational support in place (e.g., DSA, reasonable adjustments, or sessions of study skills), ADHD can continue to impair academic functioning if it remains untreated [18]. In a few cases it can be hard to tell if ADHD with or without co-occurring learning disorders or mental health symptoms, including intellectual giftedness, are different facets of the same condition or reflect separate disorders [168]. For instance, a student with undiagnosed ADHD who keeps performing badly academically, despite studying extra hard, may start to worry excessively or feel like a failure and then become depressed. This student may seek help because they are feeling anxious or depressed, but in fact the underlying condition is ADHD.

There are effective screening tools for anxiety, depression and substance misuse that can be used with university students with ADHD. The 10-item Kessler Psychological Distress Scale (K10) can be used to screen for anxiety and depression [125], or the 16-item Penn State Worry Questionnaire (PSWQ) can be used to screen for pathological worry, which is a dominant feature in generalised anxiety disorder [126]. There are useful screening tools in the appendices of the Improving Access to Psychological Therapies (IAPT) manual, including the Generalised Anxiety Disorder scale (GAD-2, GAD-7), Panic Disorders Severity Scale (PDSS), and the Patient Health Questionnaire (PHQ-9, for depression) [128]. The Simple Screening Instrument for Substance Abuse (SSI-SA) (Center for Substance Abuse Treatment, 1994) is widely used as a brief screen by practitioners and assessors with little experience of substance misuse [127]. NICE clinical guidance [CG123] also offers very clear advice and guidance for screening common mental health disorders, and recommends that if a practitioner conducting the screen identifies a possible anxiety disorder or depression, and they are not competent to perform a full mental health assessment, then they must refer the student to an appropriate healthcare professional [169].

Some students may have additional problems related to a SpLD (e.g., dyslexia) or ASD. Useful screeners for these conditions are the Adult Dyslexia Checklist which is available for free from the British Dyslexia Association website [124], and the Autism-Spectrum Quotient (AQ-10), is also available for free download [123]. If a student with ADHD screens positive for a SpLD or ASD, then a shared decision with the student can be made about the usefulness or value of a referral for a diagnostic assessment of these comorbid conditions. It might be for example, that a positive screen of either condition and careful questioning about functional impairments, will be enough to assess their impact on studying and how best to mitigate them with additional support (e.g., counselling, specialist mentoring, academic coaching, extra writing time for examinations). There is also evidence which suggests that once the core symptoms of ADHD are treated, problems related to co-occurring SpLDs, ASD traits, anxiety or depression may in turn improve [9, 158, 170]. During the shared decision-making process, an agreement with the student can be also reached about whether to include results of a positive screen for a SpLD and/or ASD in their diagnostic report, which can include a write-up about the potential complexities these conditions might add to a student’s ability to study effectively. Further details and weblinks for the screening tools are provided in Table 8.

At present there are no neuroimaging, genetic, neurochemical, or neuropsychological diagnostic tests for ADHD that are sufficiently sensitive or specific. Neuropsychological tests such as Stop Signal Reaction Time, IQ, or various computerised tests of executive functions (e.g., CANTAB) or QB-Test, can however, complement a diagnostic assessment for ADHD and provide additional information about cognitive performance [171]. Some authors (e.g., Brown [98], conceptualise ADHD as a disorder of executive function (EF), and many learning problems that university students with ADHD experience may be due to deficits in EF (e.g., poor organisation, planning and time management skills, inattention, or emotional lability) [172]. Although these EF deficits are not well reflected in cognitive performance tests [173], an assessment of EF behaviours such as those captured by the BRIEF questionnaire are strongly related to ADHD and associated functional impairments [174]. The recommendation of the expert group (and all national/international guidelines) is that a diagnostic assessment for adults with ADHD should be based on self-reported symptoms, which are best obtained by using a semi-structured in-depth diagnostic interview. An example of such a tool is the “Diagnostic Interview for Adult ADHD” (DIVA-5), which is based on the symptom and impairment criteria of the DSM-5 [129]. The ACE+ is another diagnostic tool that can be useful, and it has the option to use either DSM-5 or ICD-11 diagnostic criteria [130]. The DIVA-5 is available for a one-off fee of 10 Euro whereas the ACE+ is free to download, with digital versions in English and other languages (see Table 8 for further details and weblinks). Collateral information can also be obtained from informants such as close friends or relatives, and school records, especially for the evaluation of age of onset.

ADHD in adults is diagnosed when 5 or more symptoms of inattention and/or hyperactivity-impulsivity are present, and with several of them being present before 12 years old. These core symptoms must have persisted for at least 6 months, and in clinical practice the expectation is of a chronic trait-like course from the age of onset during childhood or early adolescence. The symptoms of ADHD should be to a degree that is inconsistent with the developmental level for that individual and must cause functional impairments in 2 or more settings (e.g., at home, university, work, with friends or relative, or in other activities) [6]. During the diagnostic process conducting a detailed evaluation of how the student’s presenting symptoms impact on their academic productivity is essential. Potential education-related impairments due to ADHD are listed in Table 9. Individually assessing and writing about education-related problems in the student’s diagnostic report will help practitioners working in student disability services to devise personalised support, as well as allow for the effectiveness of this support to be evaluated. The Weiss Functional Impairment Rating Scale – Self Report (WFRIS-S), is a useful tool for assessing and monitoring changes in functional impairments associated with ADHD in different domains [131].

**Table 9:** Potential education related problems and reasonable adjustments

Potential education related problems due to	Potential reasonable adjustment
Mind wandering (*daydreaming, intrusive task-unrelated thoughts*)	25–50% extra writing time in examinations
Poor working memory (*may need more time more time to process information and/or to understand complex conceptual ideas*)	25–50% extra writing time in examinations, separate room for writing examinations
Disorganisation and inefficiency	Academic coaching, being invigilated in an examination by a support worker familiar with ADHD
Difficulties with planning ahead, misjudging how long tasks take to perform (*different conception of time*)	25–50% extra writing time in examinations, flexible start times for an examination
Procrastination/ waiting until the “last minute” to submit work, “pulling all-nighters” (*may need more time to complete tasks*)	Academic coaching/ specialist mentoring, 10 to 20 min of a rest break during examinations
Forgetfulness (*losing things needed for university* e.g.*, student ID card, missing lectures, classes, or appointments*)	Having the ability to negotiate extensions to deadlines for assignments/ course work
Difficulty sustaining attention (*especially when bored, not engaged, or not stimulated*)	Academic coaching/ specialist mentoring, the ability to study part-time or to defer examinations
Difficulties following long explanations, note taking, or reading a lot	Academic coaching/ specialist one-to-one study skills support or subject specific support
Hyper-focus on topics of self-interest to the detriment of other topics and tasks	Academic coaching/ specialist one-to-one study skills support, subject specific support

Practitioners and assessors need to be aware that ADHD symptoms and functional impairments present differently in each student and their impact can also change over the course of their programme of studies [19]. The experience of the expert group is that some students meeting diagnostic criteria for ADHD may not want to take prescribed medication in the first instance. But as their programme of studies progresses this may change, and the student may want and require medication to reduce core symptoms of the disorder. While psychoeducation, and environmental modifications (including reasonable adjustments) can help support university students with ADHD (and may be sufficient in some cases), only medication has been found to reduce core symptoms [89]. It is the experience of the expert group that university students with ADHD often have well developed compensatory strategies such as being overly organised, almost in an obsessive manner, or studying extra hard for long periods of time to ensure adequate performance. They may also have lost the usual structured support of parents and school when they were younger, so that impairments can increasingly accrue as their course develops. During diagnostic assessments, some students can find it hard to remember what their ADHD symptoms and impairments may have been like during childhood. When this happens, it is best to focus on their presenting symptoms and establish whether at least 5 or more of them are currently present and cause impairment, then track back in time to establish as far as possible an age at which current symptoms started.

In most cases of ADHD an individual is unable to identify a clear age of onset and they have the perception that the symptoms were always present. A typical response is that the symptoms have been present for as long as they can recall. Remembering symptomatic behaviours in childhood is especially hard when the student’s parents or other care givers have given them a lot of support during their academic career, or provided them with structure and routine, or when the student, had predominantly inattentive symptoms in childhood, that were not noticed either by their parents or teachers. This is more likely in females (and some men) with ADHD, who tend to present with predominantly inattentive symptoms and few hyperactive-impulsive symptoms or less disruptive behaviour [50, 138, 175]. The gender bias in ADHD seems to become less skewed in adulthood when women with ADHD may be diagnosed, often for the first time [138]. Practitioners and assessors conducting a diagnostic assessment need to be aware that female students can present with study related problems due to ADHD for the first time whilst at university. These students may or may not have a previous diagnosis of another mental health condition, which will need to be reviewed if they are diagnosed with ADHD [138].

During face-to-face diagnostic assessments, compensatory strategies can be minimised. For instance, the student may not recognise that sustaining attention or organisation is problematic for them, when a more objective appraisal suggests that this is a persistence problem. This can occur because symptoms of ADHD reflect lifelong traits, or because the student has well developed compensatory strategies. When this happens, it’s best to assess the degree of effort that the student needs to put into maintaining a compensatory strategy (for example, *if the student did not put in extra effort to be organised then what would happen*?). Students with severe ADHD may be easier to screen and diagnostically assess, but if these students have developed good compensatory strategies (as discussed in the section on intellectual giftedness), it can be hard to determine how severe and impairing their ADHD symptoms are in other functional domains (e.g., social relationships). It may also be at a time when compensatory strategies are sufficient to mitigate ADHD related impairments, but this may not always be the case as their programme of studies progresses. Some students may present with “subthreshold symptoms” of ADHD (i.e., symptoms just below the threshold for a diagnosis of ADHD to be made), yet they appear to be significantly impaired by these symptoms and therefore need additional support, and perhaps treatment. The experience of the expert group is that impairments are also informed by co-morbidities and that several sub-threshold comorbidities (particularly of neurodevelopmental disorders) can be more impactful than a single disorder above the diagnostic threshold [176].

### Recommendation 6. Screening tools and diagnostic assessments

The expert group recommends that practitioners and assessors be given training in how to screen for and diagnostically assess ADHD using robust and evidence-based rating scales, screening tools, and standardised clinical interviews. This training should include how to conduct a detailed evaluation of education related functional impairments, write up a diagnostic report with recommendations for reasonable adjustments and make a direct referral for medical treatment if requested, to a suitably qualified practitioner with expertise in the management and treatment of ADHD in adults (e.g., a psychiatrist or mental health nurse/pharmacist non-medical prescriber). A list of standardised screening and diagnostic tools are presented in Table 8 below.

### What pharmacological and non-pharmacological interventions are useful?

Following initial psychoeducation about ADHD and its impact, NICE guidance [50] recommends making “environmental modifications”. In the context of university students with ADHD environmental modifications can take the form of “reasonable adjustments” to programmes of study under the Equality Act 2010. Potential learning problems associated with ADHD and potential reasonable adjustments are listed in Table 9. Adjustments can also be made to study environments (e.g., making available a quiet study room in the library, recommend taking frequent breaks when studying, breaking down daily targets, using digital diaries and reminders, regular forms of exercise) [172]. If these adjustments/ modifications have been applied and functional impairments continue in at least one domain (e.g. academic performance, or studying/learning difficulties), then medication should be considered.

#### Medication

NICE guidance [50] recommends psychostimulant medication (i.e., methylphenidate or lisdexamphetamine) as first-line medical treatment for ADHD in adults. Psychostimulant medications are among the most effective medications in use within adult mental health [89], and among the most efficacious of all common medical drugs [177]. Stimulant medications often produce a substantial reduction in ADHD symptoms and associated impairments. However, stimulant medications can also have adverse effects, which in most cases are either dose-related, mild, or transient such as headache, reduced appetite, nausea, palpitations, difficulty falling asleep and dry mouth [89]. In a few cases, these adverse effects may be undesirable, and an individual may decide to stop using stimulant medication. Stimulant medications can also increase blood pressure and heart rate, therefore people who take these medications are assessed at baseline and monitored during their treatment [50]. Empirical research about the efficacy of treating university students with ADHD is rare and the extent to which prescribers consider the unique demands of university life when prescribing medication to students is unknown [178].

The first randomised controlled trial of lisdexamphetamine with a sample of 24 university students diagnosed with ADHD was conducted by DuPaul et al., [179]. In this study, lisdexamphetamine was administered over a 5-week period and large reductions in the students ADHD symptoms were observed, alongside improvements in their task management, planning, organisation, use of study skills and working memory. Although the short duration of this study precluded an assessment of academic functioning over the long-term, in other studies, university students with ADHD who took medication did report improvements in their note taking, scores on tests, writing output and completion of course work [180]. In a pharmaco-epidemiological study from Sweden young people with ADHD taking medication were also found to have better scores in standardised university entrance examinations when compared to peers with ADHD not taking medication [181]. It is noted, however, that a substantial number of university students with ADHD do not take their medication as prescribed [182]. Some university students with ADHD may use their medication flexibly, with optimum dosing during times of writing assignments or studying for examinations and then no medication on days without academic work, e.g., at weekends or during holidays [183]. When treating university students with ADHD, prescribing practitioners therefore need to be open to discussing the benefits and drawbacks of flexible dosing with students and be willing to offer appropriate guidance and advice [184, 185].

#### Non-pharmacological interventions

The view of the expert group is that non-pharmacological interventions are particularly important for university students who want or need to learn how to best manage their ADHD and overcome the learning difficulties that they experience. Medication alone maybe sufficient for a subgroup of university students, but persistent difficulties are more often seen, and additional support maybe required. Non-pharmacological interventions begin with psychoeducation. The experience of the expert group is that newly diagnosed students are keen to have a conversation about their diagnosis, including whether or not to disclose it to academic staff or future employers, the benefits, and drawbacks of taking medication, including flexible dosing, “drug holidays”, effects of medication on alcohol or other drugs, the positive attributes of ADHD (e.g., creativity), psychological interventions and reasonable adjustments. Research about the effectiveness of non-pharmacological interventions for adults with ADHD is mixed and inconclusive, but positive effects have been reported for mindfulness on core symptoms of ADHD including mind wandering [186], dialectical behaviour therapy (DBT) and cognitive behavioural therapy (CBT) [187–189].

Although research about non-pharmacological interventions for university students with ADHD is limited, new studies have been published. For instance, Anastopoulos et al. [190] and Eddy et al. [191] reported on the findings of a randomised controlled trial (RCT) that examined the efficacy of a CBT based program called ACCESS (Accessing Campus Connections and Empowering Student Success) for university students with ADHD. During the ACCESS program - psychoeducation, cognitive and behavioural strategies targeting executive function (EF) and patterns of maladaptive thinking, were delivered. Participants, who met DSM-5 diagnostic criteria for ADHD and were taking medication, were recruited from two large public universities in the USA and randomly assigned to either the ACCESS program group (*n* = 119) or a Delayed Treatment Control (DTC) group (*n* = 131). The findings revealed that the ACCESS program group participants self-reported significant improvements in their knowledge of ADHD, symptoms of inattention, EF, utilisation of disability accommodations (or reasonable adjustments), as well as a moderate decline in maladaptive thinking, when compared to DTC group participants. However, neither ACCESS program and DTC group participants reported significant improvements in their interpersonal functioning and educational outcomes (grade point average/GPA and course grade completion). The authors concluded that the ACCESS program made a large difference to the participants core symptoms of ADHD and EF.

Indeed, as noted previously, EF deficits have been shown to mediate the association between ADHD and impairments in academic functioning [100]. The finding that the ACCESS program positively impacted on the participants EF is therefore encouraging. It also supports the findings of an earlier pilot study about a CBT based group intervention to enhance EF functioning in university students with ADHD [172], and strengthens a more recent finding about how *steep* temporal discounting may play a key role in the daily life challenges that university students with ADHD encounter. Temporal discounting (TD) describes how the subjective value of a reward significantly declines when the said reward is delayed [192]. In a pilot study by Scheres and Solanto [193], *steep* TD was not only associated with combined type ADHD, specifically the hyperactivity-impulsivity symptom domain, but also with poor utilisation of learning and/or study skills. TD was therefore postulated to be an important target for EF interventions for university students with or without ADHD [193], more so for interventions that were designed to activate and sustain motivation to pursue a long-term goal for a reward, such as pursuing and completing a university degree [194]. Findings like this could be useful for enhancing the effectiveness of CBT based interventions for university students with ADHD like the ACCESS program, by for example, tailoring EF interventions to also target TD. Maybe this could improve educational outcomes and perhaps interpersonal functioning of university students with ADHD, which in the study reported by Anastopoulos et al. [190] showed no significant improvements.

The report that the ACCESS program made a large difference to the students’ core symptoms of ADHD, seems to contradict what the World Federation of ADHD international consensus statement acknowledged about good treatments for ADHD being available, but even the best treatments are only partially effective [164]. Overall, there is only low-quality evidence that CBT interventions might be beneficial for treating core symptoms of ADHD in adults, in the short-term, or for improving co-occurring symptoms of anxiety and depression [164, 195]. It was noted by Anastopoulos et al., [190], that participants in both study groups increased their use of ADHD medications over the course of the study. Perhaps this was the real reason that the participants core symptoms of ADHD improved. After all, this is what ADHD medications are designed to do and treatments for ADHD usually become more effective when medication is combined with a CBT intervention [195], or when multimodal interventions are used [196].

Hence academic coaching, which tends to be a derivative of CBT, could be another useful intervention for optimising coping strategies in university students with ADHD. For instance, coaching has been used to help identify study goals, develop study plans and strategies for achieving these plans, monitoring their progress towards attaining them and to foster self-determination [197]. In one study, academic coaches helped university students with ADHD to develop better time management, organisational skills, pay more attention in classes and to take good notes, and improvements in these skills were observed after 8 weeks [198]. In another study, university students with ADHD reported that academic coaching had helped to enhance their self-discipline, self-efficacy, study skills, ability to formulate realistic goals and to think more about long-term goals and maintain motivation to achieve them [199]. Additional benefits of coaching can be in helping university students with ADHD feel more in control of their emotions and behaviours in the face of external demands [200]. Academic coaching (or specialist mentoring, or specialist one-to-one study skills support), can also be funded via DSA as specialist access and learning facilitators (Band 4). Academic coaching, supportive counselling and/or CBT, whether delivered face-to-face or online can be effective non-pharmacological interventions for university students with ADHD [188, 189, 201], and the potential of these interventions to improve academic performance is evident in the promising results of recent studies e.g. [172, 190].

### Recommendation 7. Multimodal interventions

The expert group recommends multimodal interventions for university students with ADHD, that comprise a variety of interventions including environmental modifications, psychoeducation, medication, academic coaching, DBT, CBT, counselling and/or mindfulness-based interventions. University counselling and disability services do tend to offer a range of psychosocial interventions for students, whether delivered online, face-to-face or in a group.

### What are the staff training and developmental needs?

In the Institute for Employment Studies report to the Office for Students, practitioners working in university disability services identified a need for training and development to enable them to both support university students with ADHD and the academic staff working with them [52]. The SpLD Assessments and Standards Committee (SASC) [167], also recommended that practitioner psychologists and specialist teacher assessors require appropriate training to identify “*specific learning difficulties and patterns of behaviour that together would strongly suggest that a student has ADHD*” (p.11). The need for staff training and development was discussed during the consensus meeting, and it included training in how to liaise with and refer university students with ADHD to a suitably qualified practitioner for a diagnostic assessment (e.g., a psychiatrist, mental health nurse/ pharmacist non-medical prescriber). Practitioners and assessors seemed keen to receive “certified training” as a way to achieve the SASC recommendations for “appropriate training”. A certified educational programme about ADHD at university level 6 or 7, could be developed and delivered for example online, as a post-qualification professional training or continuous professional development (CPD). But at present, no such course/programme exists in the UK. UKAAN offers training for healthcare professionals and can deliver bespoke training to practitioners and assessors who work with university students, and some disability services have already done so. During the consensus meeting some practitioners and assessors said they often gained relevant experience by having previously worked, or currently working, with university students with ADHD or through their own personal lived experiences, and that they made use of these experiences in their role.

### Recommendation 8. Training and development

The expert group recommends that staff training, and development be prioritised under the inclusive practice agenda in higher education. This training should include psychoeducation, procedures for screening and assessing for ADHD, and useful strategies for supporting university students with ADHD. This will enhance the knowledge and skills of practitioners and assessors who work with and/or support university students with ADHD.

## Discussion & conclusion

This was a report of the UKAAN expert consensus meeting about university students with ADHD, which was held before the COVID-19 pandemic. Since then, the pandemic has altered higher education in a monumental way. When lockdown was first imposed in the UK, university campuses were suddenly closed. Students and staff had to quickly adapt to online delivery of lectures and classes, and there was uncertainty about being able to access digital technologies and quite places to study or work at home. There was also confusion among students about study expectations, assessments, workloads, retention, and completion [202–204]. Undoubtedly the pandemic has caused much suffering, frustration, fear, loss and other negative thoughts, emotions, and experiences for many people, including university students with ADHD [205]. However, findings about the impact of the pandemic on university students has been mixed. Frampton and Smithies [206], reported on a Students Minds survey about life during the pandemic involving 1100 university students. The findings of this survey revealed that 74% of respondents reported that the pandemic had a negative impact on their mental health and wellbeing, whilst only 10% of respondents reported positive effects. In this survey, disabled and non-disabled students were also asked whether they agreed or disagreed with the statement “*online learning has allowed me to engage with my course more positively*”, and the findings revealed that 59% of disabled students compared with 55% of non-disabled students disagreed with the statement. This also suggests that just under-half of these students agreed with the statement. In another study, 79 university students in one Faculty of Life Sciences were surveyed and participated in focus groups about how they experienced the sudden shift to online learning during the lockdown [207]. This study found that 75% of the students who participated in the study, reported that their life had become more difficult and 50% reported that learning outcomes would be hard to achieve, but after 12 weeks into the lockdown, corresponding rates changed to 57 and 71% respectively [207].

The findings of existing studies do suggest that during the COVID-19 lockdown, virtual learning for some university students may have had benefits such as enabling greater attendance, engagement, and participation in teaching sessions, especially for students who previously felt anxious about asking questions in front of others or some disabled students [202]. Students who were used to spending time online – on the Internet including social media platforms for example, seemed to exhibit strong motivation for eLearning, and reported lower levels of distress during the pandemic [208]. However, there are also concerning reports about ADHD being a risk factor for COVID-19 infection [209, 210]. These reports are perhaps pertinent for university students with ADHD who may have participated in demonstrations during the pandemic such as Black Lives Matter (BLM), living arrangements in student halls of residence, sexual harassment, assault and “rape culture” in UK universities [206, 211], or illegal COVID raves [212], or the COVID anti-vaccine and lockdown protests [213]. It can be argued that the pandemic may have longer-term negative consequences on current and future career prospects for university students with ADHD, but outside of this, no firm conclusions from the existing research can be drawn.

Evidence is stronger for poor education (or academic) performance and achievement having a long-term negative impact on mental health, wellbeing, and socio-economic outcomes [214]. Even though there is a paucity of research about university students with ADHD in the UK and rest of Europe, the importance of attending to the mental health of university students in the UK has been recognised. The Royal College of Psychiatrists recently published a college report on the mental health of higher education students, and Sedgwick-Müller et al., contributed a section on ADHD in this report [1]. The expert group is also aware that ADHD is a hidden disability within UK HEIs and its categorisation as a SpLD may be contributing to this, therefore university students with ADHD continue to be at risk of marginalisation and disadvantage. The expert group recommends that ADHD should be catered for under a separate category within UK HEIs, as this may enable greater recognition of ADHD and for its impact on learning within higher education to be adequately assessed and mitigated. With aspirations towards widening participation and inclusive practices in higher education [52], understanding exactly “what works” best for university students with ADHD is imperative. The four key stages in a student’s lifecycle are access to higher education *(the extent to which students can gain entrance to different types of HEIs),* retention *(the likelihood of continuing or withdrawing from a programme of studies),* attainment *(the extent to which university students are enabled to achieve their full academic potential)*, and progression *(successful transitions within a programme of studies and afterwards into employment or further study*)” [215], p.5). Each of these 4 key stages in a student’s lifecycle can be adversely affected by either having and/or not recognising ADHD, and by delaying access to a screening, diagnostic assessment, treatment, and educational support. Interventions in a student’s first year at university, according to Clery and Topper, should focus on enhancing their academic achievement because retention, attainment, and progression tends to be more favourable for university students who perform well academically in their first year [216].

In summary, UKAAN convened an expert consensus meeting to provide an informed understanding about the impact of ADHD on the educational (or academic) outcomes of university students and to highlight an urgent need for timely access to treatment and management. An overview of key issues, as well as expert advice and guidance has been offered. In Table 10 below, the main recommendations of the expert group are summarised. There is little doubt that university students with ADHD are struggling with long delays in accessing a diagnostic assessment, treatment, and personalised educational support. The provision of rapid access treatment and care pathways can be challenging for clinicians working in specialist NHS ADHD clinics, but examples of good practice are also beginning to emerge, with some university disability services drawing on their own budgets to support their students. Further work is needed to develop and evaluate efficient and cost-effective treatment and care pathways for university students with ADHD (for example see Fig. 4), and to adopt models of best practice across the sector. University students, including those with ADHD, are at a crucial transitioning stage in life and their success at university is likely to determine their success in highly competitive employment markets. This strengthens the argument to support all university students in an inclusive manner. Methods for inclusive teaching and learning are also likely to cater to disabled students, including university students with ADHD.

**Table 10:** Summary of the main recommendations from the expert group

**1.** ADHD can cause problems learning, but it should not be subsumed under the category of a specific learning (disability) or difference (SpLD) within HEIs. A separate category for ADHD will enable a prevalence estimate among university students to be established. It may also lead to greater recognition of ADHD within higher education, personalised packages of support and further research to examine the impact of ADHD on educational outcomes.	
**2.** University students who present to disability services with complaints of learning problems that seem related to SpLDs, DCD or ASD should also be screened for ADHD.	
**3.** University students with anxiety and depression who report persisting learning problems should also be screened for ADHD.	
**4.** Programmes of staff training and development about ADHD, should include psychoeducation and raise awareness about the potential stigma that university students or others may perceive, including its consequences and potential impact on disclosure, the screening and diagnostic process, treatment, and support.	
**5.** Rapid access treatment and care pathways for university students with ADHD should be developed collaboratively between central support services (e.g. disability services), NHS primary and secondary care, or private providers. The university disability services fund for SpLD diagnostic assessments should also become available to students who require a diagnostic assessment for ADHD.	
**6.** Practitioners and assessors in higher education should be trained in how to screen and diagnostically assess ADHD, conduct a detailed evaluation of education related functional impairments, write up a diagnostic report with recommendations for reasonable adjustments and make a direct referral to a suitably qualified practitioner with expertise in assessing comorbidities and treating ADHD.	
**7**. Multimodal interventions comprising psychoeducation, environmental modifications (e.g. reasonable adjustments, disabled students’ allowance, DSA), medication, academic coaching, DBT and/or CBT, counselling, mindfulness, are useful for university students with ADHD.	
**8.** Staff training, and development should be prioritised under the inclusive practice agenda in higher education. This training is likely to enhance knowledge about how best to support university students with ADHD and up-skill practitioners and assessors who work with these students.	

## Data Availability

Data sharing is not applicable to this article as no data sets were generated or analysed during the study.

## Abbreviations

ACE: ADHD Child Evaluation
AHEAD: Association for Higher Access & Disability
ADHD: Attention Deficit Hyperactivity Disorder
APA: American Psychiatric Association
AQ-10: Autism-Spectrum Quotient
ASD: Autism Spectrum Disorder
ASRS: Adult ADHD Self-report Rating Scale
CADDRA: Canadian ADHD Practice Guidelines
CBT: Cognitive Behavioural Therapy
DIVA-5: Diagnostic Interview for ADHD in adults
DBT: Dialectical Behavioural Therapy
DSA: Disabled Students Allowance
DCD: Developmental co-ordination disorder
DSM-5: Diagnostic and Statistical Manual of Mental Disorders version 5
EL: Emotional lability
EF: Executive Functions
HEIs: Institutions of Higher Education
HESA: The Higher Education Statistical Authority
IAPT: Improving Access to Psychological Therapies
IES: Institute of Employment Studies
MDD: Major Depressive Disorder
MD: Math Disability
NHS: National Health Service
NICE: National Institute for Health and Care Excellence
OfS: Office for Students
PDSS: Panic Disorders Severity Scale
PHQ-9: Patient Health Questionnaire
PS: Processing Speed
PSWQ: Penn State Worry Questionnaire
QbTest: Quantified Behavior Test
RD: Reding Disability
SEND: Special Educational Needs and Disabilities
SEN: Special Educational Needs
SLD: Specific Learning Disorders
SASC: SpLD Assessment and Standards Committee
SpLDs: Specific learning differences
SSI-SA: The Simple Screening Instrument for Substance Abuse
UKAAN: UK Adult ADHD Network
UK: United Kingdom of Great Britain and Northern Ireland
WAIS: Wechsler Adult Intelligence Scale
WFRIS-S: Weiss Functional Impairment Rating Scale – Self Report
WHO: World Health Organisation
WM: Working Memory
WD: Writing Disability
